# Arterio-venous metabolite and electrolyte responses to low-load training with and without blood flow restriction versus high-load training to failure

**DOI:** 10.1007/s00421-025-06067-8

**Published:** 2025-12-08

**Authors:** Sanghyeon Ji, Michaela Vicas, Alexander Franz, Tobias Boemer, Michael Behringer, Patrick Wahl

**Affiliations:** 1 Section Exercise Physiology, German Sport University Cologne, Cologne, Am Sportpark Müngersdorf 6, 50933 Cologne, Germany; 2 The German Research Center for Elite Sport, Cologne, Germany; 3 Department of Adult Reconstruction, ATOS Orthoparc Clinic Cologne, Cologne, Germany; 4 Department of Orthopedics and Trauma Surgery, University Hospital Bonn, Bonn, Germany; 5 Department of Trauma and Orthopedic Surgery, BG Klinikum Ludwigshafen, Ludwigshafen, Germany; 6 Department of Sports Sciences, Goethe University Frankfurt, Frankfurt, Germany

**Keywords:** Metabolic stress, Mechanical stress, Occlusion training, Hyperkalemia, Arterial-venous blood analysis

## Abstract

**Purpose:**

Low-load resistance training with blood flow restriction (BFR) has gained popularity for eliciting muscular adaptations comparable to high-load resistance training. However, its acute metabolic and electrolyte responses within the exercising limb, particularly under exhaustive conditions, remain insufficiently characterized. This study aimed to assess these responses using simultaneous arterial and venous blood sampling during unilateral elbow flexion to volitional failure under three conditions: low-load (LL-RT, 30%1RM), low-load with BFR (LL-BFR-RT, 30%1RM, 50%LOP), and high-load (HL-RT, 75%1RM).

**Methods:**

Ten healthy men (26.8 ± 4.6 years) completed all exercise conditions in a randomized cross-over design. Catheters were placed in the radial artery and antecubital vein of the exercising arm. Serum creatine kinase (CK) and lactate dehydrogenase (LDH) were assessed as indirect muscle damage markers.

**Results:**

LL-RT produced the highest total workload (692 ± 251 kg), exceeding both LL-BFR-RT (378 ± 58.7 kg) and HL-RT (327 ± 65.1 kg, *p* < 0.001). Muscle pain perception assessed using a visual analog scale increased during exercise, with a highest level in LL-BFR-RT (*p* < 0.01). LL-BFR-RT also induced the most pronounced venous perturbations (e.g., reduced pH and sO_2_, elevated pCO_2_ and K^+^), while arterial responses remained modest across conditions. CK increased slightly at 48 h post-exercise across all conditions (*p* = 0.036), while LDH was highest following HL-RT (*p* < 0.001).

**Conclusion:**

These findings suggest that LL-BFR-RT to failure induces substantial local metabolic and ionic stress within the exercising limb despite reduced mechanical loading. The marked venous disturbances, alongside minimal increases in systemic damage markers, support its use as a metabolically potent yet mechanically efficient training modality when applied with care.

## Introduction

In recent decades, resistance training with blood flow restriction (BFR) has attracted growing interest as an effective training method with application across a wide range of populations and settings (Lixandrão et al. 2018; Hughes et al. 2017; Pignanelli et al. 2021; Ferguson et al. 2021). This training approach involves applying an inflatable cuff or elastic band around the proximal region of the exercising limb, typically in combination with low mechanical loads (LL-BFR-RT, e.g., 30% of one-repetition maximum, 1RM). The applied pressure leads to a partial restriction of arterial inflow and an almost occlusion of venous outflow, creating a hypoxic environment and promoting the accumulation of metabolites within the working muscle (Patterson et al. 2019). Despite the reduced mechanical loading, LL-BFR-RT has been shown to induce muscle hypertrophy comparable to that achieved with conventional high-load resistance training (HL-RT, e.g., 75% 1RM), although it typically fails to produce equivalent strength gains (Lixandrão et al. 2018). Moreover, LL-BFR-RT has been reported to promote mitochondrial and microvascular adaptations, thereby contributing to improvements in muscle endurance capacity (Vissing et al. 2020).

While the exact mechanisms underlying these adaptations are not yet fully understood, existing studies on the acute physiological responses to LL-BFR-RT have proposed several contributing factors: metabolite accumulation (Fujita et al. 2007; Nakajima et al. 2018), increased muscular activation /recruitment (Yasuda et al. 2014), and elevated post-exercise growth hormone levels (Fujita et al. 2007). These factors are thought to stimulate muscle protein synthesis and other signaling pathways, thereby contributing to physiological adaptations associated with BFR training (Pignanelli et al. 2021; Loenneke et al. 2010). In particular, metabolic perturbations (e.g., pronounced acid-base imbalance, lactate accumulation) appear to play an important role in driving both muscular and microvascular adaptations (Pignanelli et al. 2021; Loenneke et al. 2010). Although previous research has provided valuable insights into the physiological effects of BFR exercise, most studies have primarily focused on systemic and/or indirect (non-invasive) measurements, offering limited information about the physiological changes occurring locally within the occluded limb. This compartment may represent a partially isolated and metabolically distinct environment. In particular, a comprehensive characterization of metabolic, ionic, and oximetric dynamics within the arterial and venous systems of the exercising limb remains largely unexplored. Such data may offer unique insights into local tissue stress and regulatory processes, as arterial and venous compartments reflect different physiological functions – oxygen supply vs. metabolite accumulation/clearance. Understanding their dissociation could improve our interpretation of localized muscular stress during BFR exercise.

A large number of previous studies comparing LL-BFR-RT with conventional resistance training have employed volume-matched protocols with fixed repetition schemes (e.g., 30-15-15-15). While this methodological approach allows for standardized and controlled comparisons with regard to the total workload, it may obscure differences in the internal physiological demands of the respective training modalities (Franz et al. 2020, 2024). Indeed, our previous study showed that this fixed-repetition protocol resulted in almost complete exhaustion during LL-BFR-RT, with some participants unable to complete the prescribed repetitions, which was not the case for performing the same protocol with free-flow low-load resistance training (LL-RT) (Franz et al. 2020). Consequently, the physiological responses observed under LL-BFR-RT may be confounded by differing levels of muscular fatigue when compared to free-flow control conditions, complicating the interpretation of BFR-specific effects (Franz et al. 2020, 2024; Kolind et al. 2023).

From a clinical and practical perspective (e.g., conservative therapy, rehabilitation), it is essential to assess the additional strain potentially imposed by BFR training, particularly regarding excessive muscular stress and damage, as well as metabolically induced disruptions in electrolyte homeostasis. Exercise-induced disturbances in ionic balance, such as elevated extracellular potassium concentrations (i.e., hyperkalemia), may be exacerbated under local hypoxia and metabolic acidosis typically associated with BFR exercise. Transient elevations in extracellular potassium can alter cardiac membrane excitability and conduction velocity (Weiss et al. 2017). While such responses are usually well tolerated in healthy individuals, they may pose risks for those with compromised cardiovascular function, where even short-lived hyperkalemia could increase their risk of arrhythmias or other adverse cardiac events (Franz et al. 2020; Weiss et al. 2017). Although our previous study, which used a workload-matched protocol, found no significant difference in ionic responses between LL-RT and LL-BFR-RT in young, healthy individuals (Franz et al. 2020), it is unclear whether more pronounced ionic shifts might occur when resistance exercise is performed to volitional failure. A better understanding of acute ionic shifts in response to BFR training, especially under exhaustive conditions, may help clarify the safety profile of BFR and contribute to more individualized and risk-adjusted protocols. Beyond safety considerations, exercise-induced ionic perturbations appear to be potent molecular stressors that promote adaptations in muscle ionic homeostasis (e.g., increased Na^+^-K^+^-ATPase expression), thereby enhancing fatigue resistance and exercise performance (Christiansen 2019). Investigating the acute ion dynamics in response to exhaustive resistance training under various loading conditions, with and without BFR, may provide further insight into how ionic disturbances related to modulated mechanical (load) and/or metabolic (BFR) stress. Such insights could help clarify how acute ionic perturbations interact with mechanical and metabolic stress and potentially contribute to subsequent adaptations.

Therefore, the present study aimed to investigate acute local responses to resistance exercise performed to volitional failure under three different conditions: low-load resistance training (LL-RT), LL-RT with BFR (LL-BFR-RT), and high-load resistance training (HL-RT). Specifically, we assessed metabolic, ionic, and oxygenation-related changes, along with serum markers of muscle damage and inflammation. To capture compartment-specific responses, we applied invasive arterial and venous blood sampling within the exercising (and occluded) limb, allowing for detailed characterization of local physiological perturbations across different mechanical loading conditions, with and without BFR.

## Method

### Participants

Ten healthy males (age: 26.8 ± 4.59 years, height: 171 ± 7.60 cm, body mass: 79.0 ± 8.00 kg) participated in this study. All participants had at least two years of resistance training experience (≥ 2 sessions per week) but had no prior exposure to systematic BFR training before the start of the investigation. All participants were fully informed about the experimental procedures and possible risks and provided their informed consent by signing a document. The study was approved by the local Ethics Committee of the University Hospital Duesseldorf (Trial-ID: 2015104498) and was performed according to the Declaration of Helsinki.

### Study design

To investigate arterial and venous metabolic and ionic responses, as well as potential exercise-induced muscle damage under varying mechanical loads and with the application of BFR, a randomized cross-over design was employed. Each participant completed a pre-testing session followed by three experimental test sessions on separate days. During the pre-testing, the individual concentric 1RM of the elbow flexors (bicep curl) in the self-reported dominant arm was determined using a dumbbell, following a previously described protocol (Jessee et al. 2017). Participants completed 6–8 unilateral elbow flexion attempts, starting with an estimated load of 60–75% of their maximum. Testing was conducted with participants standing upright against a wall, elbow fully extended, forearm supinated, and the contralateral arm placed behind the back to minimize extraneous movement. The load was progressively increased with each attempt until the participant failed to lift a load heavier than the last successful attempt. A 2-min rest interval was provided between attempts. The heaviest weight successfully lifted with a full range of motion was defined as the 1RM.

**Fig. 1:**
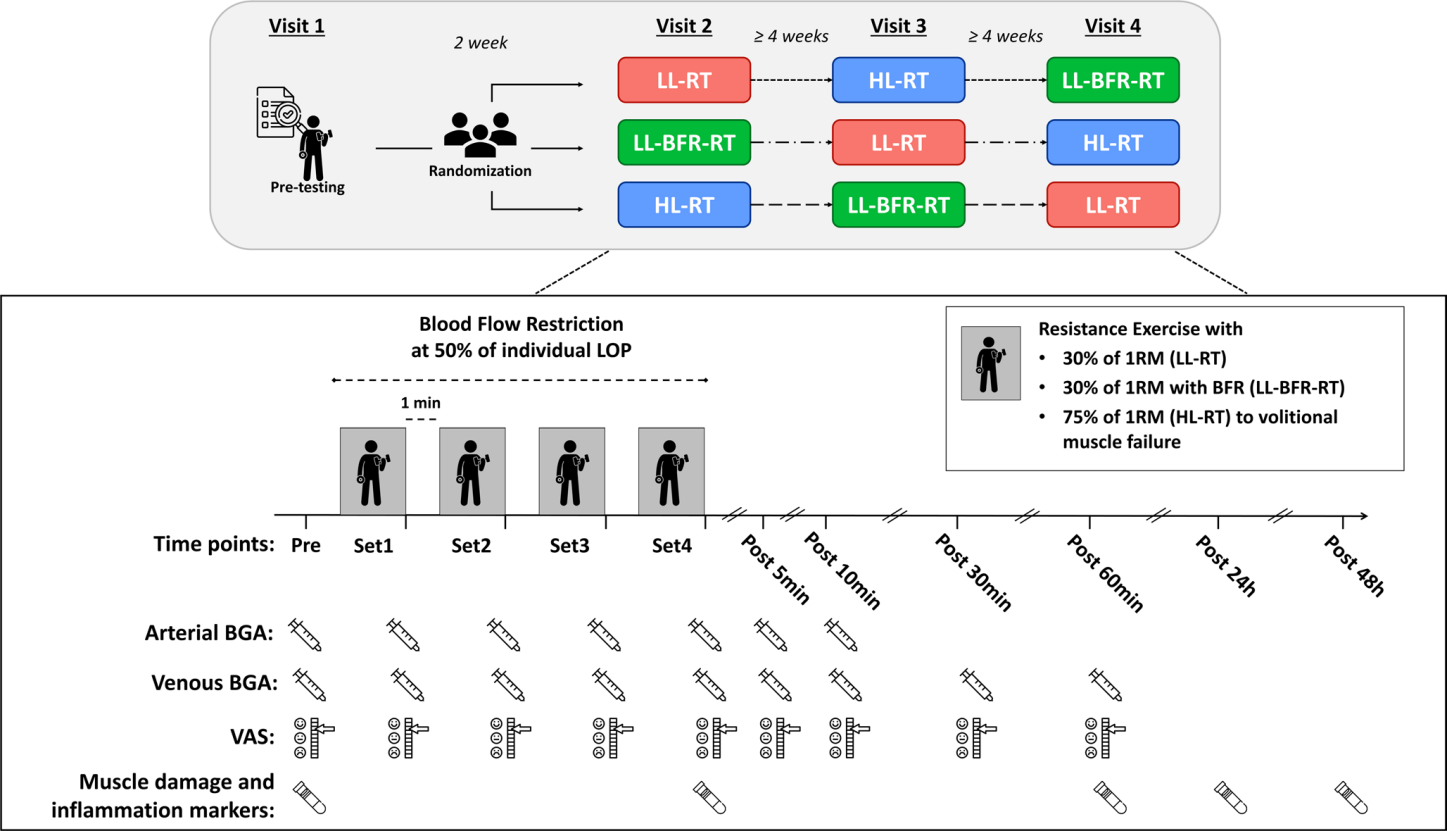
Schematic overview of the randomized cross-over study design, depicting three exercise trials in various sequences and the experimental timeline, including the measurement time points. In addition to the pre-testing session, all participants performed three experimental test sessions, separated by at least four weeks, consisting of low-load (LL-RT), LL-RT with blood flow restriction (LL-BFR-RT), or high-load resistance exercise (HL-RT) to volitional muscle failure. LOP = individual arterial limb occlusion pressure; 1RM = individual one repetition maximum; BGA = blood gas analysis; VAS = visual analog scale

Following a 2-week rest period, participants completed the first of three experimental sessions, each involving the elbow flexor exercise protocol under one of three conditions: LL-RT, LL-BFR-RT, or HL-RT. In order to reduce the impact of the repeated bout effect (Hyldahl et al. 2017), the experimental sessions were separated by 4 weeks of rest. Follow-up measurements took place at 24 and 48 h after each experimental session. All participants were instructed to avoid consuming alcohol for at least 24 h and to refrain from vigorous physical activity for 48 h before each experiment session. A schematic overview of the experimental protocol and measurement time points is provided in Fig. 1.

### Sample size calculation

Based on our previous study on the metabolic and ionic changes during BFR exercise (Franz et al. 2024), we assumed a moderate to high effect size (*η*^*2*^_*p*_ = 0.05–0.54) with respect to our main outcomes from blood gas analysis. To determine the necessary sample size, we conducted a power analysis using G*Power (version 3.1.9.7). With assumptions of a mean effect size (cohen’s *f*) of 0.4, an alpha error (ɑ) of 0.05, and statistical power (1–*β*) of 0.80, the analysis determined a minimum sample size of *N* = 9 for a repeated measures design (within-between interaction (3 condition x 9 time points), correlation level among repeated measures: 0.50, non-sphericity correction: 1.0). Consequently, the attained sample size of *N* = 10 is considered sufficient to detect the expected metabolic and ionic responses across exercise conditions.

### Exercise trials

All experimental sessions consisted of unilateral biceps curls with the dominant arm, using a dumbbell (0.25 kg increments, ScSports, Emmerich, Germany). In each session, participants completed one of the following three experimental loading protocols (in a randomized order): (1) LL-RT: 30% of the 1RM, (2) LL-BFR-RT: 30% of the 1RM with BFR, (3) HL-RT: 75% of the 1RM. Each protocol consisted of four sets performed to volitional muscle failure, with 60 s of rest between sets.

Throughout the exercise, participants maintained an upright standing position with their back against a wall, ensuring continuous elbow contact with the wall throughout the movement. Each repetition involved lifting the dumbbell to full elbow flexion (~ 130°), with a total duration of four seconds–two seconds for both the concentric and eccentric phases – and no static transition period between contractions. The tempo was controlled by a metronome (60 beats·min^− 1^). Each set was stopped when voluntary muscle failure occurred, defined as the inability to maintain the prescribed tempo. Based on the performed repetitions in each set, the total workload (repetitions × applied mechanical load [kg]) was determined for comparison between exercise trials.

The LL-BFR-RT trial was performed using 50% of the individual arterial limb occlusion pressure (LOP; 82.5 ± 8.7 mmHg), which is within the commonly recommended range for the safe and effective BFR application during resistance training (Patterson et al. 2019). Recent evidence suggests that cuff pressures ≥ 50% LOP are sufficient to accelerate fatigue or amplify the metabolic stress required for adaptation. Much higher pressures (80% LOP) tend to increase perceived discomfort/pain (Cerqueira et al. 2021; Das and Paton 2022; Lixandrão et al. 2018). Further considering the continuous application of BFR during exercise, we selected a cuff pressure of 50% LOP as a pragmatic compromise, aiming to elicit a sufficient physiological stimulus while preserving tolerability throughout the exhaustive four-set protocol. Pilot testing confirmed that this setting was well tolerated and minimized the risk of premature termination due to excessive discomfort. The individual LOP was determined before the training session with an inflatable tourniquet of 11.5 cm width (Easi-Fit Contour, Delfi Medical Inc., Vancouver, Canada), which was placed proximal on the exercising arm. Following a 10-min rest period, participants lay in a supine position, while cuff pressure was gradually increased by an automated system with an internal pressure sensor (PTSII, Delfi Medical Inc., Vancouver, Canada) until arterial blood flow in the radial artery was no longer detectable. This pressure was recorded as the individual LOP. Before the start of the LL-BFR-RT trial, the cuff was inflated using a pneumatic inflator (PTSII, Delfi Medical Inc., Vancouver, Canada) and maintained throughout the entire exercise period. It was deflated one minute after completion of the protocol–immediately following blood sampling after the fourth set.

### Arterial and venous blood sampling and analysis

To assess metabolic and ionic changes during the exercise, arterial and venous catheters were inserted into the trained arm prior to the exercise protocol. Participants were positioned in a lying posture on a standard medical examination table. Under local anesthesia with lidocaine hydrochloride, arterial access was established via radial artery puncture, while venous access was obtained through a dorsal hand vein (Rete venosum dorsale manus) using Seldinger technique (Franz et al. 2020).

Blood gas analysis (BGA) samples were collected using a 2 mL syringe anticoagulated with 50 IU balanced lithium heparin. Arterial blood was sampled at the following time points: pre-exercise, between each of the four exercise sets, and at 0-, 5-, and 10-min post-exercise. Venous blood was sampled at the same time points, with additional samples taken at 30- and 60- min post-exercise. Prior to each sampling, approximately 5 ml of blood was initially collected from the catheters with a single-use syringe and discarded. The obtained BGA samples were analyzed directly using an automated blood gas analyzer (GEM premier 3500, Werfen, Bedford, USA). The following parameters were measured for both arterial and venous samples: pH, partial pressures of carbon dioxide (pCO_2_) and oxygen (pO_2_), concentration of lactate (La^−^), potassium (K^+^), sodium (Na^+^), calcium (Ca_2_^+^), bicarbonate (HCO_3_^−^), and oxygen saturation (sO_2_).

To assess changes in indirect markers for exercise-induced muscle damage and inflammation, 8.5 mL of venous blood was collected using a vacutainer blood withdrawal system (Becton Dickinson, Heidelberg, Germany) at pre-exercise, immediately post-, and at 60 min, 24 h, and 48 h post-exercise. After storage at 7 °C for ~ 30 min for deactivation of coagulation factors, the blood samples were centrifuged for 10 min at 3000 rpm and 4 °C (EBA200, Hettich, Mühlheim, Germany). Serum was then stored at – 80 °C until subsequent analysis. Concentrations of creatine kinase (CK) and lactate dehydrogenase (LDH) (U·L^− 1^) were determined using an enzymatic-photometric method on an automated analyzer (ADVIA 1800, Siemens Healthcare, USA).

### Subjective pain assessment

Participants reported their perceived muscle pain in the exercising arm using a 100 mm visual analog scale (VAS), where 0 cm indicated “no pain” and 10 cm indicated “maximal pain”. The VAS was obtained immediately before and after each exercise set, as well as at 5-, 10-, 30-, and 60-min post-exercise.

### Statistical analysis

Statistical analyses were conducted using R (version 4.2.2). Homoscedasticity and normality were visually assessed through residual and Q-Q plots. To compare changes in measures over time, linear mixed models (*lme4* package) were applied, incorporating time (4 to 9 levels) and exercise condition (3 levels: LL-RT, LL-BFR-RT, and HL-RT) as fixed effects. To account for inter- and intra-individual variability, each participant’s baseline measure was included as a random intercept. For total repetitions and workload, a mixed-effects model with one-way ANOVA was used, treating participants as a random effect factor and condition as a fixed factor to evaluate differences between exercise protocols. In case of a significant main and/or interaction effect, multiple pairwise post-hoc comparisons with Bonferroni correction were performed (*emmeans* package) to determine which factor levels differ significantly from one another. To estimate the practical relevance, effect size values (partial eta squared, *η*^*2*^_*p*_) were calculated for the interaction and main effects. According to Cohen (2013), a *η*^*2*^_*p*_ ≥ 0.01 indicates small effects, ≥ 0.059 medium effects, and ≥ 0.138 large effects. An alpha level of 0.05 was interpreted as statistically significant. Data are expressed as the mean ± standard deviation. In addition, mean differences (MDs) with 95% confidence intervals (CIs) were reported where relevant.

## Results

**Fig. 2:**
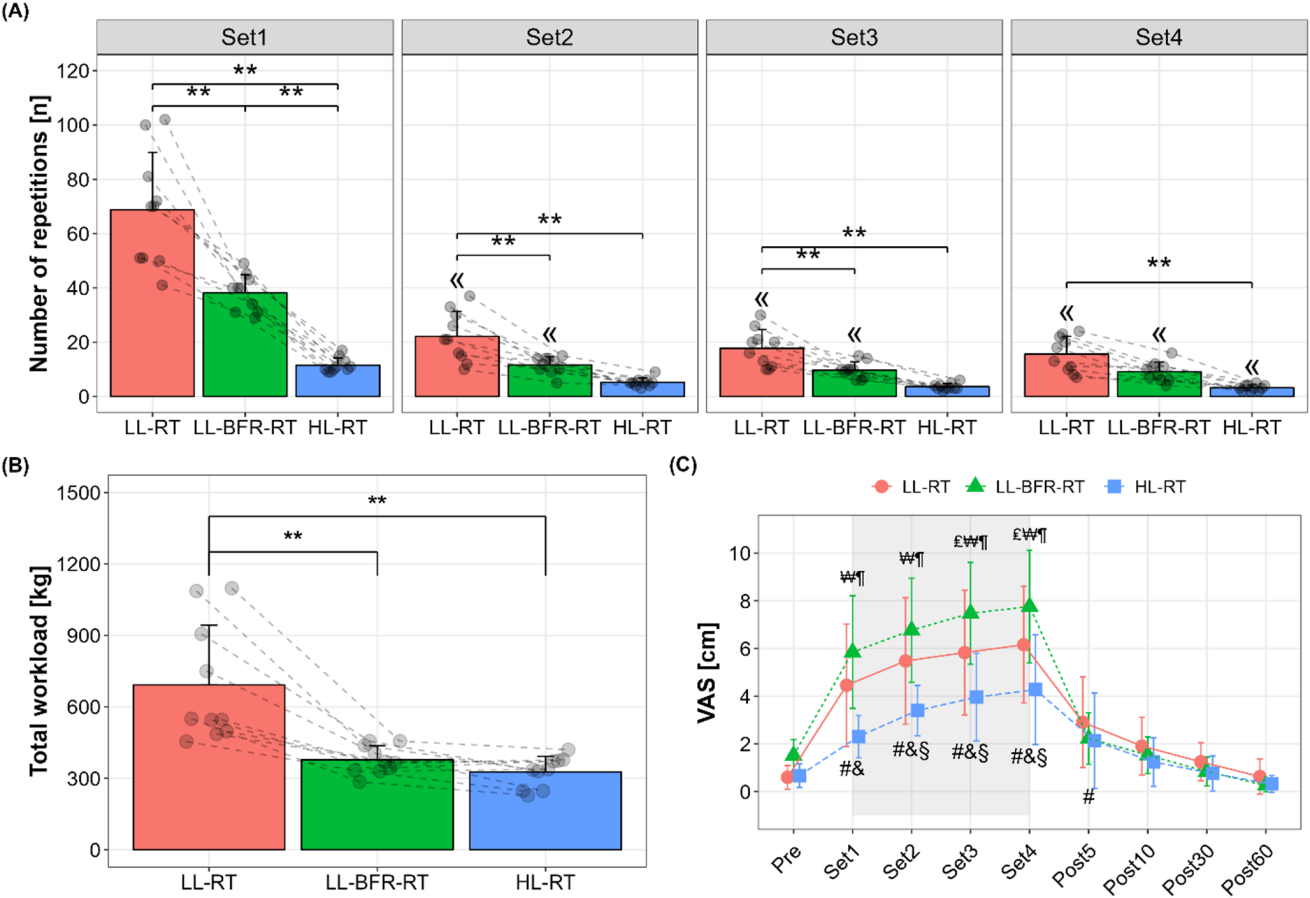
Number of repetitions (**A**), total workload (**B**), and subjective muscle pain measured by visual analog scale (VAS) (**C**) in low-load resistance exercise (LL-RT), LL-RT with blood flow restriction (LL-BFR-RT), and high-load resistance exercise (HL-RT). ^«^Significantly different from Set1 within the respective condition (*p* < 0.05), ^#^significantly different from Pre within LL-RT (*p* < 0.01), ^&^significantly different from Pre within LL-BFR-RT (*p* < 0.001), ^§^significantly different from Pre within HL-RT (*p* < 0.001), ^₤^significant difference between LL-RT and LL-BFR-RT (*p* < 0.05), ^₩^significant difference between LL-RT and HL-RT (*p* < 0.05), ^¶^significant difference between LL-BFR-RT and HL-RT (*p* < 0.001), ^**^significant difference between conditions (*p* < 0.05)

For the number of repetitions performed during each exercise trial (Fig. 2A), a significant interaction was found (*p* < 0.001, *η*^*2*^_*p*_ = 0.61). Post-hoc comparisons showed that participants consistently performed a significantly higher number of repetitions in the LL-RT trial compared to other trials (*p* < 0.05), except for LL-BFR-RT in Set 4 (*p* = 0.10). When comparing the LL-BFR-RT and HL-RT trials, a significant difference in the number of repetitions was observed only during Set 1 (*p* < 0.001). When repetitions were summed across all sets, participants achieved the highest total repetitions in LL-RT (124 ± 40 reps), followed by LL-BFR-RT (69 ± 12 reps) and HL-RT (24 ± 5 reps), with significant differences between all conditions (*p* < 0.001, *η*^*2*^_*p*_ = 0.86). Regarding the total workload (Fig. 2B), there was a significant condition effect (*p* < 0.001, *η*^*2*^_*p*_ = 0.72), with LL-RT (692 ± 251 kg) being significantly higher than LL-BFR-RT (378 ± 58.7 kg) and HL-RT (327 ± 65.1 kg, *p* < 0.01 for both), while LL-BFR-RT and HL-RT did not differ (*p* = 1.00).

Regarding subjective muscle pain measured by VAS (Fig. 2C), a significant time x condition interaction was observed (*p* < 0.001, *η*^*2*^_*p*_ = 0.20). Post-hoc analyses showed that muscle pain intensity increased across the exercise sets and returned to baseline levels 5 min post-exercise. Comparisons between conditions revealed LL-RT and LL-BFR-RT induced a more pronounced increase in muscle pain intensity compared to HL-RT (*p* < 0.01), with LL-BFR-RT exhibiting significantly higher levels than LL-RT in Sets 3 and 4 (*p* ≤ 0.05).

**Fig. 3:**
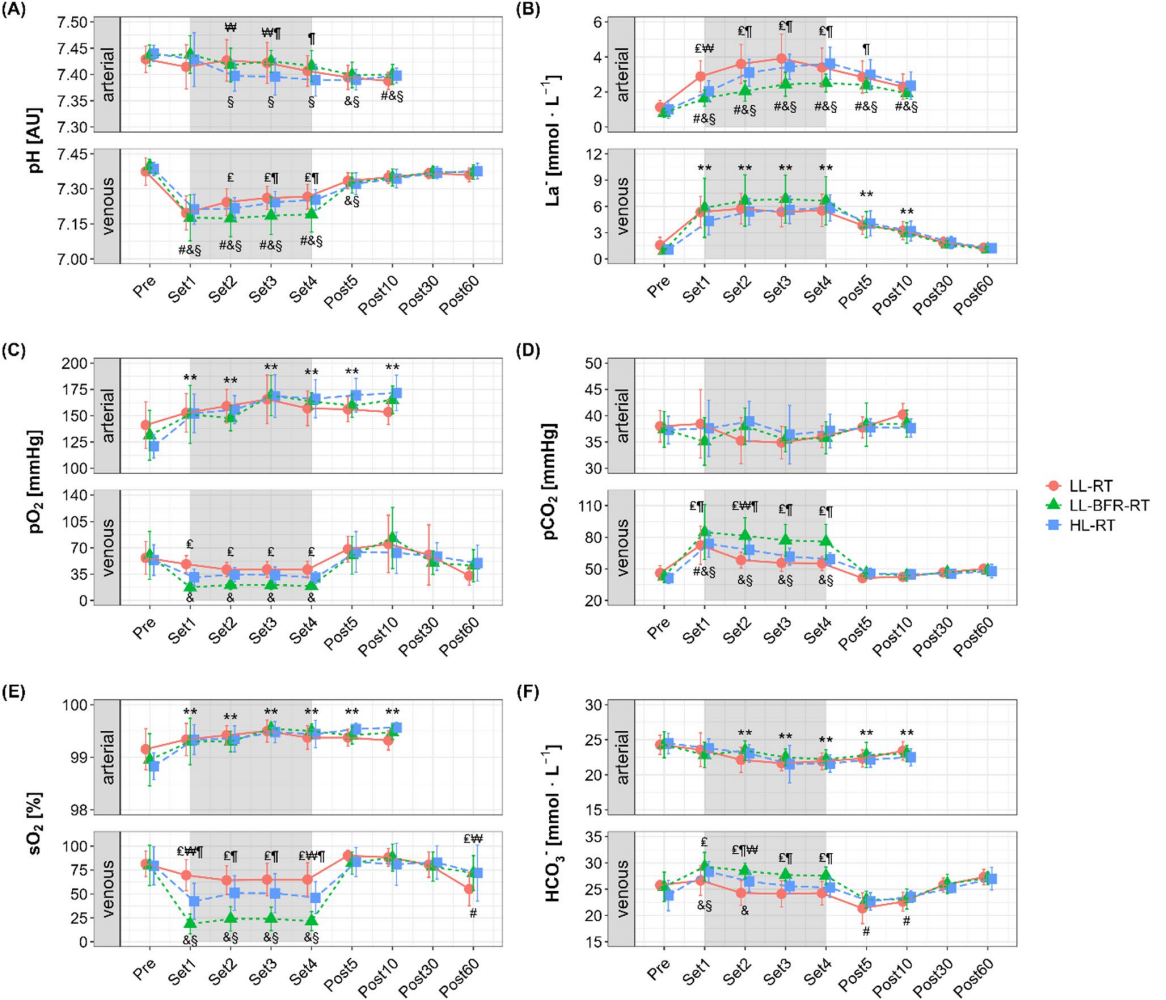
Changes in arterial and venous blood gas and oximetry parameters in response to low-load resistance exercise (LL-RT), LL-RT with blood flow restriction (LL-BFR-RT), and high-load resistance exercise (HL-RT). **A** pH, **B** lactate (La^−^), **C** oxygen partial pressure (pO_2_), **D** carbon dioxide partial pressure (pO_2_), **E** oxygen saturation (sO_2_), **F** bicarbonate (HCO_3_^−^). ^#^significantly different from Pre within LL-RT (*p* < 0.001), ^&^significantly different from Pre within LL-BFR-RT (*p* < 0.001), ^§^significantly different from Pre within HL-RT (*p* < 0.05), ^₤^significant difference between LL-RT and LL-BFR-RT (*p* < 0.05), ^₩^significant difference between LL-RT and HL-RT (*p* < 0.001), ^¶^significant difference between LL-BFR-RT and HL-RT (*p* < 0.001), ^**^significantly different from Pre (*p* < 0.001, main time effect across conditions)

Changes in blood gas and oximetry parameters measured in the arterial and venous systems in response to each exercise trial are shown in Fig. 3.

A significant time x condition interaction was observed for both arterial (*p* = 0.03, *η*^*2*^_*p*_ = 0.11) and venous pH (*p* < 0.001, *η*^*2*^_*p*_ = 0.14) (Fig. 3A). Post-hoc analyses revealed that arterial pH decreased only in HL-RT (Sets 2–4, *p* < 0.001 vs. Pre), with significantly lower values compared to LL-RT in Sets 2–3 (*p* ≤ 0.03) and compared to LL-BFR-RT in Sets 3–4 (*p* ≤ 0.03). Venous pH decreased significantly during exercise across all trials (*p* < 0.001 vs. Pre), with LL-BFR-RT exhibiting a more pronounced decrease compared to LL-RT (Sets 2–4, average MD = -0.07 AU, 95% CI [-0.12 to -0.03 AU], *p* < 0.001) and HL-RT (Sets 3–4, average MD = -0.06 AU, 95% CI [-0.11 to -0.01 AU], *p* < 0.01).

Arterial La^−^ (Fig. 3B) showed a significant interaction (*p* < 0.001, *η*^*2*^_*p*_ = 0.17), with LL-RT (Sets 1–4) and HL-RT (Set 2–Post 5) inducing larger increases compared to LL-BFR-RT (*p* ≤ 0.03). Venous La^−^ showed only a significant main effect of time (*p* < 0.001, *η*^*2*^_*p*_ = 0.72), increasing during exercise and remaining elevated post-exercise across all trials (*p* < 0.001 vs. Pre). No significant condition (*p* = 0.06, *η*^*2*^_*p*_ = 0.02) or interaction effects (*p* = 0.17, *η*^*2*^_*p*_ = 0.08) were observed for venous La^−^ (*p* ≥ 0.06).

Arterial pO_2_ (Fig. 3C) showed a significant main effect of time (*p* < 0.001, *η*^*2*^_*p*_ = 0.37), with increasing levels during exercise that remained elevated after exercise across all trials (*p* < 0.001 vs. Pre). No significant condition (*p* = 0.49, *η*^*2*^_*p*_ = 0.01) or interaction effects (*p* = 0.09, *η*^*2*^_*p*_ = 0.09) were observed for arterial pO_2_. Venous pO_2_ showed a significant interaction (*p* = 0.001, *η*^*2*^_*p*_ = 0.12), with a greater decrease during exercise in LL-BFR-RT compared to LL-RT (Sets 1–4, average MD = -23.5 mmHg, 95% CI [-44.0 to -2.95 mmHg], *p* ≤ 0.05), but no significant difference between HL-RT and LL-RT (*p* ≥ 0.08) or between HL-RT and LL-BFR-RT (*p* ≥ 0.32).

Arterial pCO_2_ (Fig. 3D) showed a main effect of time (*p* = 0.006, *η*^*2*^_*p*_ = 0.09) but remained unchanged during the exercise sets across all trials (*p* ≥ 0.57 vs. Pre), with no significant condition (*p* = 0.57, *η*^*2*^_*p*_ = 0.01) or interaction effects (*p* = 0.13, *η*^*2*^_*p*_ = 0.09). Venous pCO_2_, exhibited a significant interaction (*p* < 0.001, *η*^*2*^_*p*_ = 0.21), with LL-BFR-RT inducing the most pronounced increase during exercise compared to the other trials (Sets 1–4, average MD vs. LL-RT = 19.4 mmHg, 95% CI [9.69 to 29.1 mmHg], *p* ≤ 0.007; average MD vs. HL-RT = 14.2 mmHg, 95% CI [4.47 to 23.8 mmHg], *p* ≤ 0.02).

For arterial sO_2_ (Fig. 3E), a significant main effect for time was observed (*p* < 0.001, *η*^*2*^_*p*_ = 0.37), with levels increasing during the exercise sets that remained elevated post-exercise across all trials (*p* < 0.001 vs. Pre). No significant condition (*p* = 0.99, *η*^*2*^_*p*_ < 0.001) or interaction effects (*p* = 0.11, *η*^*2*^_*p*_ = 0.09) were observed for arterial sO_2_. Venous sO_2_ showed a significant interaction (*p* < 0.001, *η*^*2*^_*p*_ = 0.36), with LL-BFR-RT inducing the most pronounced decrease during exercise compared to other conditions (Sets 1–4, average MD vs. LL-RT = -44.0%, 95% CI [-59.1 to -28.4%], *p* < 0.001; average MD vs. HL-RT = -25.5%, 95% CI [-40.6 to -10.4%], *p* < 0.001).

Arterial HCO₃^−^ (Fig. 3F) revealed a main effect of time (*p* < 0.001, *η*^*2*^_*p*_ = 0.28), decreasing during the exercise sets and remaining post-exercise across all trials (*p* ≤ 0.004 vs. Pre), with no significant condition (*p* = 0.23, *η*^*2*^_*p*_ = 0.02) or interaction effects (*p* = 0.22, *η*^*2*^_*p*_ = 0.08). Venous HCO_3_^−^ showed a significant interaction (*p* < 0.001, *η*^*2*^_*p*_ = 0.15), with LL-BFR-RT inducing the most pronounced increase, exceeding LL-RT across all exercise sets (average MD = 3.43 mmol⸱L^− 1^, 95% CI [1.35 to 5.50 mmol⸱L^− 1^], *p* < 0.001) and HL-RT in Sets 2–4 (average MD = 2.12 mmol⸱L^− 1^, 95% CI [0.05 to 4.20 mmol⸱L^− 1^], *p* ≤ 0.03).

**Fig. 4:**
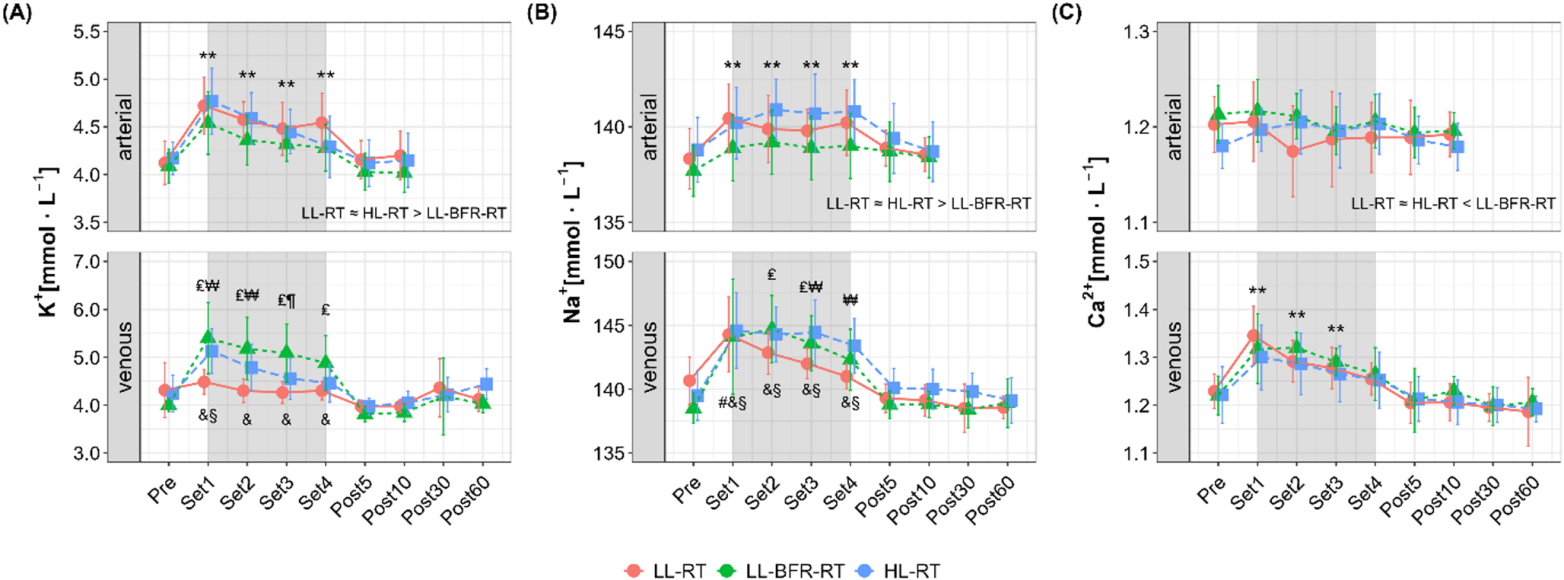
Changes in arterial and venous electrolyte concentrations in response to low-load resistance exercise (LL-RT), LL-RT with blood flow restriction (LL-BFR-RT), and high-load resistance exercise (HL-RT). **A** Potassium (K^+^), **B** sodium (Na^+^), and **C** calcium (Ca_2_+). ^#^significantly different from Pre within LL-RT (*p* < 0.001), ^&^significantly different from Pre within LL-BFR-RT (*p* < 0.001), ^§^significantly different from Pre within HL-RT (*p* < 0.05), ^₤^significant difference between LL-RT and LL-BFR-RT (*p* < 0.05), ^₩^significant difference between LL-RT and HL-RT (*p* < 0.001), ^¶^significant difference between LL-BFR-RT and HL-RT (*p* < 0.001), ^**^significantly different from Pre (*p* < 0.001, main time effect across conditions), main condition effect across time points is depicted in individual figures, < (*p* < 0.05) and ≈ (*p* > 0.05)

Changes in electrolyte concentrations in both arterial and venous systems in response to each exercise trial are shown in Fig. 4.

For arterial K^+^ (Fig. 4A), significant main effects for time (*p* < 0.001, *η*^*2*^_*p*_ = 0.54) and condition (*p* < 0.001, *η*^*2*^_*p*_ = 0.12) were observed. Further analyses revealed that arterial K^+^ increased during the exercise sets (*p* < 0.001 vs. Pre) and was higher in LL-RT and HL-RT compared to LL-BFR-RT (both *p* < 0.001). Venous K^+^ showed a significant interaction (*p* < 0.001, *η*^*2*^_*p*_ = 0.23). Post-hoc analyses indicated that LL-BFR-RT (Sets 1–4) and HL-RT (Set 1) induced significant increases in venous K^+^ during the exercise sets (*p* < 0.001 vs. Pre), with higher levels compared to LL-RT (LL-BFR-RT: Sets1–4, average MD = 0.81 mmol⸱L^− 1^, 95% CI [0.32 to 1.31 mmol⸱L^− 1^], *p* ≤ 0.01; HL-RT: Sets 1–2, average MD = 0.58 mmol⸱L^− 1^, 95% CI [0.09 to 1.07 mmol⸱L^− 1^], *p* ≤ 0.03).

Arterial Na^+^ (Fig. 4B) revealed significant main effects of time (*p* < 0.001, *η*^*2*^_*p*_ = 0.27) and condition (*p* < 0.001, *η*^*2*^_*p*_ = 0.18). Post-hoc analyses indicated that arterial Na^+^ increased during the exercise sets (*p* < 0.001 vs. Pre) with higher levels in LL-RT and HL-RT compared to LL-BFR-RT (both *p* < 0.001). For venous Na^+^, we found a significant interaction effect (*p* = 0.017, *η*^*2*^_*p*_ = 0.11). Post-hoc analyses showed significant increase during Set 1 in all exercise trials (*p* < 0.001), while LL-BFR-RT and HL-RT continued to show higher levels in the following sets (*p* ≤ 0.01 vs. Pre). Consequently, venous Na^+^ was significantly higher in LL-BFR-RT (Sets 2–3, average MD = 2.03 mmol⸱L^− 1^, 95% CI [0.01 to 4.05 mmol⸱L^− 1^], *p* ≤ 0.04) and HL-RT (Sets 3–4, average MD = 2.63 mmol⸱L^− 1^, 95% CI [0.66 to 4.61 mmol⸱L^− 1^], *p* ≤ 0.01) compared to LL-RT.

Arterial Ca^2+^ (Fig. 4C) showed a significant condition effect (*p* = 0.005, *η*^*2*^_*p*_ = 0.05), with higher levels in LL-BFR-RT compared to the other trials (*p* ≤ 0.03). No significant time (*p* = 0.13, *η*^*2*^_*p*_ = 0.05) or interaction effects (*p* = 0.26, *η*^*2*^_*p*_ = 0.07) were observed for arterial Ca^2+^. Venous Ca^2+^ showed significant main effects of time (*p* < 0.001, *η*^*2*^_*p*_ = 0.55) and condition (*p* = 0.05, *η*^*2*^_*p*_ = 0.02). Post-hoc analyses revealed increased venous Ca^2+^ during Sets 1–3 (*p* < 0.001 vs. Pre) with no significant difference between conditions (*p* ≥ 0.09).

As summarized in Table 1, there were significant time and condition effects for CK and LDH, respectively. Serum CK concentration increased significantly at 48 h post-exercise across all protocols compared to baseline level (*p* = 0.04), while LDH levels were generally higher in HL-RT compared to the other conditions (*p* < 0.001).

**Table 1:** Serum Creatin kinase (CK) and lactate dehydrogenase (LDH) concentrations in response to low-load resistance exercise (LL-RT), LL-RT with blood flow restriction (LL-BFR-RT), and high-load resistance exercise (HL-RT)

	Condition	Pre	Post	Post 1 h	Post 24 h	Post 48 h	*p*-values from mixed effect model / η^2^_p_		
							Time	Condition	Time × Condition
CK [U·L^− 1^]	LL-RT	182 ± 90.6	200 ± 90.9	193 ± 80.5	218 ± 96.9	267 ± 155	**0.036 / 0.07** (Pre < Post 48 h)	0.163 / 0.03	0.372 / 0.06
	LL-BFR-RT	160 ± 77.4	199 ± 98.4	198 ± 96.9	199 ± 104	215 ± 122			
	HL-RT	178 ± 62.7	239 ± 72.9	251 ± 76.4	227 ± 90.5	213 ± 38.5			
LDH [U·L^− 1^]	LL-RT	216 ± 31.5	203 ± 30.9	212 ± 30.1	234 ± 80.5	192 ± 25.7	0.158 / 0.05	**< 0.001 / 0.14**	0.609 / 0.04
	LL-BFR-RT	191 ± 37.5	209 ± 56.6	201 ± 35.2	205 ± 37.4	199 ± 20.2			
	HL-RT^*#^	228 ± 31.5	241 ± 40.9	266 ± 78.2	248 ± 89.3	221 ± 49.2			

*Significant difference to LL-RT (*p* < 0.001), ^#^significant difference to LL-BFR-RT (*p* < 0.001)

## Discussion

This study aimed to investigate metabolic, ionic, and electrolyte responses to resistance exercise under different mechanical loading conditions (LL-RT and HL-RT), and with the additional application of BFR (LL-BFR-RT), by analyzing both arterial and venous compartments of the exercising limb. Performing the exercise protocol to muscle failure resulted in a higher total workload in LL-RT compared to HL-RT and LL-BFR-RT (Fig. 2B). Despite the lower mechanical load, LL-BFR-RT elicited greater venous metabolic stress–indicated by increased La⁻ and pCO_2_ levels and reduced pH–than both free-flow conditions (Fig. 3). In terms of electrolyte shifts in the venous system, the responses to LL-BFR-RT and HL-RT were more pronounced than those to LL-RT (Fig. 4). In contrast, arterial responses during LL-BFR-RT remained attenuated, likely reflecting localized accumulation due to restricted clearance (Figs. 3 and 4). Importantly, none of the exercise protocols induced meaningful increases in systemic markers of muscle damage or inflammation (Table 1).

Previous studies have shown that BFR blunts the exercise-induced hyperemia by limiting arterial blood flow (Downs et al. 2014; Mladen et al. 2025). It has been suggested that this reduction in perfusion may be compensated by an increased oxygen extraction to maintain muscular oxidative metabolism (Jones et al. 2012; Salzmann et al. 2021). Our data support this compensatory mechanism: while arterial pO_2_ and sO_2_ remained stable or slightly increased during LL-BFR-RT, similar to free-flow conditions, venous pO_2_ and sO_2_ decreased substantially, more than under free-flow conditions. In addition to the increased oxygen extraction from arterial blood, the restricted venous outflow likely impaired the clearance of deoxygenated blood and metabolites, thereby further intensifying local metabolic stress (Staunton et al. 2015; Takano et al. 2005; Kolind et al. 2023; Franz et al. 2020). The resulting metabolic perturbations–especially accumulation of CO_2_ and H^+^–may have further enhanced oxygen diffusion to the working muscles caused by a rightward shift of the oxygen dissociation curve (i.e., Bohr effect) (Sarelius and Pohl 2010; Salzmann et al. 2021), thus supporting the compensatory mechanism during the initial phase of exercise. However, throughout the exercise, the ongoing blood flow restriction likely intensified local hypoxia (blood stasis-induced microvascular hypoxia) (Salzmann et al. 2021) and limited oxidative energy production. This may have led to a growing reliance on anaerobic metabolism, as reflected by the pronounced venous acidosis (i.e., decreased pH) and elevated pCO_2_ observed during LL-BFR-RT.

Interestingly, our data revealed a marked increase in venous HCO_3_^−^ during LL-BFR-RT, a finding that appears counterintuitive given the simultaneous venous acidosis. Elevated HCO_3_^−^ concentration is typically associated with compensation for alkalosis (Hamm et al. 2015), making its concurrent rise with decreasing pH physiologically unexpected. This paradox may reflect altered acid-base dynamics specific to the occluded limb under BFR conditions. A potential explanation is that increased local metabolic activity, coupled with restricted clearance, led to CO_2_ accumulation in the venous compartment. Supporting this, LL-BFR-RT elicited a pronounced reduction in venous pO_2_ (alongside stable arterial pO_2_), indicating continued oxidative metabolism. The concurrent rise in venous pCO_2_ suggests enhanced CO_2_ production and retention, which may have driven the carbonic anhydrase-catalyzed conversion of CO_2_ to H^+^ and HCO_3_^−^ within erythrocytes (Geers and Gros 2000; Jones 2008). While this process may have contributed to elevated HCO_3_^−^, the ongoing accumulation of H^+^–from both anaerobic glycolysis and CO_2_ hydration–may have exceeded local buffering capacity, particularly that mediated by hemoglobin. This would explain the persistent venous acidosis despite increased HCO_3_^−^ levels (Jones 2008; Geers and Gros 2000). Taken together, this acid-base imbalance likely reflects a distinct characteristic of the localized metabolic stress and impaired clearance under BFR. Although speculative, these findings underscore the unique physiological environment induced by LL-BFR-RT, which constitutes a potent intra- and extracellular stressor that may trigger signaling pathways associated with structural and functional adaptations in skeletal muscle (Christiansen et al. 2019, 2020, 2021). In this context, Christiansen et al. demonstrated that BFR training improves local physiological capacities–including enhanced leg convective O_2_ transport (Christiansen et al. 2020), muscular H^+^ transport and blood H^+^-buffering capacity (Christiansen et al. 2021) as well as glucose uptake by exercising muscles (Christiansen et al. 2019)–thereby leading to improved local homeostasis during dynamic exercise and contributing to improved muscular performance. Notably, by applying a within-subject design in which each participant trained one leg with BFR and the other without, they were able to show that these adaptations originate from peripheral mechanisms–specifically, the pronounced metabolic and redox perturbations elicited by exercising under BFR–independent of mechanical loading or systemic circulation (Christiansen et al. 2019, 2020, 2021). Taken together, our data provide indirect mechanistic support for the potential of LL-BFR-RT to drive local adaptations through pronounced metabolic stress despite low mechanical loading, which may offer advantages in both athletic and rehabilitative settings (Pignanelli et al. 2021).

Repeated muscle contractions during exercise are accompanied by ionic shifts, including intracellular Na^+^ accumulation and extracellular K^+^ efflux (McKenna et al. 2008). These disturbances are normally counteracted by the Na⁺/K⁺-ATPase, which actively restores transmembrane gradients and stabilizes membrane excitability (Aronson and Giebisch 2011; Lindinger and Cairns 2021). However, the presence of metabolic acidosis, especially in the extracellular compartment, can impair the Na^+^/K^+^-ATPase function, partly due to inhibited Na^+^-H^+^-exchange activity (Fenn et al. 1958; Aronson and Giebisch 2011). This in turn, diminishes K^+^-reuptake into the cell, resulting in a net shift of K^+^ from the intracellular to the extracellular space (Juel 2008; McKenna et al. 2008). This mechanistic framework is indirectly reflected in our data, showing a pronounced accumulation of venous K⁺ during LL-BFR-RT, accompanied by marked metabolic acidosis (i.e., a pronounced decrease in venous pH) in the exercising limb. In addition, mechanically restricted venous outflow (venous pooling) likely further limited the ionic clearance, thereby contributing to the more pronounced electrolyte accumulation in the venous blood. In contrast, during free-flow conditions, although the same mechanisms of ionic imbalance and ATPase impairment associated with metabolic acidosis were likely present, the continuous / increased blood flow may have facilitated ionic clearance from the working limb (Lindinger and Cairns 2021), as reflected by higher arterial K⁺ levels but lower venous accumulation during LL-RT and HL-RT compared to LL-BFR-RT. Despite disturbed K^+^-handling, mean venous K⁺ concentrations did not reach hyperkalaemic levels (> 5.5 mmol·L^− 1^) during any of the exercise conditions in the present study. However, it is noteworthy that some participants exhibited sustained elevations in venous K⁺ levels (ranging 5.6–7.1 mmol·L⁻¹) exceeding the upper reference limit during LL-BFR-RT and HL-RT (three cases in each condition). While these results may be attributed to the exhaustive characteristic of the exercise protocol–particularly with high mechanical loading or BFR–it emphasizes the necessity for caution when applying BFR during (exhaustive) exercise in clinical populations with limited physical capacity and potential cardiovascular risk (Franz et al. 2020; Weiss et al. 2017).

In the present study, the intensified metabolic and ionic disturbances observed during LL-BFR-RT were accompanied by increased pain perception. This finding is consistent with previous reports showing that LL-RT-BFR, particularly when performed to volitional failure, elicits greater subjective pain responses compared to free-flow exercise, regardless of load (Queiros et al. 2023). While the mechanical compression of the BFR cuff itself may contribute to discomfort, accelerated metabolite accumulation and local acidosis might be major contributors to the heightened pain perception during LL-BFR-RT. Supporting this, Pollak et al. (2014) demonstrated a possible link between pain perception and metabolite accumulation during exercise by showing that intramuscular infusion of high concentrations of metabolites associated with muscle contractions (H^+^, lactate, and ATP) elicited clear pain sensations (e.g., burning and aching). In this context, we observed moderate inverse relationships between subjective pain levels and both venous pH (*r* = -0.587) and venous sO_2_ (*r* = -0.509), which aligns with findings by Cockfield et al. (2024) reporting a moderate relationship between deoxyhemoglobin levels and pain perception during arm cranking with and without BFR. Together, these results highlight the challenge in the practical application of BFR training. Although increased metabolic stress, characterized by heightened homeostatic perturbations and local hypoxia during BFR exercise (despite low mechanical loads) is considered as one of key stimuli promoting molecular signaling pathways and subsequent physiological adaptations (Loenneke et al. 2010; Ferguson et al. 2021), the accompanying pain and discomfort may be a limiting factor of BFR training, particularly in populations with low exercise tolerance or high sensitivity to exertional discomfort. Therefore, an essential objective in BFR training design is to identify the optimal balance between eliciting sufficient metabolic stress to trigger adaptive pathways and maintaining tolerable discomfort levels (Hammert et al. 2024). Adjusting the applied cuff pressure represents one potential strategy. In the present study, the cuff pressure of 50% LOP was selected as a pragmatic compromise within the range recommended for safe and effective BFR training (Patterson et al. 2019). This ensures that sufficient physiological stress is applied, while maintaining participant tolerance throughout the exhaustive four-set protocol. However, recent evidence suggests that even lower cuff pressures (20–40% LOP) can already reduce limb blood flow during exercise in a dose-dependent manner (Mladen et al. 2025). In addition, higher pressures (> 40% LOP) do not appear to further enhance muscle adaptations, but they may increase discomfort (Lixandrão et al. 2015, 2018). Beyond the cuff pressure applied, the inflation pattern–whether the cuff remains inflated continuously throughout the whole bout of exercise or is applied intermittently only during exercise sets–also influences both physiological and perceptual responses. Studies using continuous BFR protocols tend to use lower % LOP to maintain tolerability across repeated sets, whereas intermittent inflation patters often require higher % LOP to compensate for the reduced occlusion time. These interacting factors highlight the need for compressive and systematic research exploring the impact of modifying BFR exercise protocols on the acute and chronic BFR-induced adaptations. Thereby, particular attention should be given to identify the minimal effective and most tolerable combination for safe and effective training.

Despite the exhaustive nature of the exercise protocol, the present study observed only minimal increases in indirect biomarkers of muscle damage (CK and LDH), regardless of the mechanical load applied. Importantly, LL-BFR-RT did not elicit any additional inflammatory or damage-related responses compared to free-flow low- or high-load resistance exercise. These findings are consistent with previous research indicating that BFR does not amplify muscle damage responses to low-load resistance exercise, even under exhaustive conditions (Loenneke et al. 2014; Franz et al. 2024). This supports the safety profile of LL-BFR-RT and suggests that it can be applied without inducing excessive structural muscle strain in healthy individuals.

The present investigation is not without limitations, which should be considered when interpreting the findings. Generalization of the present results should be made with caution, as our data were collected from a small group of healthy, young men with experience in resistance training, which ultimately limits the translational potential for other populations, such as women, older adults, or clinical groups. Furthermore, although we determined the individual LOP under standardized conditions in a supine position to ensure methodological consistency, the training was performed in a standing position. Given the influence of body position on the measurement of LOP, potentially modulated by individual characteristics (Queiros et al. 2024), there might be a discrepancy between the intended and the actual pressure stimulus during BFR exercise, particularly with individual variations. Future studies should consider this factor by aligning LOP assessment with the exercise position to minimize potential effects.

In conclusion, the present results showed that BFR during low-load biceps curls performed to volitional failure induced intensified metabolic and ionic perturbations in the exercising limb, which were comparable to, or even exceeding, those observed during free-flow exercise with equal or higher mechanical loads. These responses were primarily reflected in the venous system, whereas changes in arterial blood were rather attenuated, underlining the importance of local perfusion dynamics and limited metabolite clearance as key mechanisms underlying the BFR exercise-specific stimulus. Although these physiological changes during BFR exercise were accompanied by increased acute pain perception, no additional impacts on systemic markers of muscle damage or inflammation were observed compared to conventional resistance exercise protocols. Taken together, LL-BFR-RT, as a metabolically demanding but mechanically less strenuous modality, could offer additional benefits compared to traditional resistance training methods. Future research should focus on optimizing BFR programming to enhance tolerability without compromising its adaptive stimulus.

## Data Availability

The data that support the findings of this study are available from the corresponding author upon reasonable request. Source data underlying all Figures and Tables are provided as a Source.

## Abbreviations

1RM: One-repetition maximum
BFR: Blood flow restriction
BGA: Blood gas analysis
Ca_2_^+^: Calcium
CK: Creatine kinase
CI: Confidence interval
HCO_3_^−^: Bicarbonate
HL-RT: High-load resistance training
K^+^: Potassium
La^−^: Lactate
LDH: Lactate dehydrogenase
LL-BFR-RT: Low-load resistance training with blood flow restriction
LL-RT: Low-load resistance training
LOP: Arterial limb occlusion pressure
MD: Mean difference
Na^+^: Sodium
pCO_2_: Partial pressures of carbon dioxide
pO_2_: Partial pressures of oxygen
sO_2_: Oxygen saturation
VAS: Visual analog scale
